# Characterization of Non-Toxigenic *Clostridioides difficile* Strains Isolated from Preterm Neonates and In Vivo Study of Their Protective Effect

**DOI:** 10.3390/jcm9113650

**Published:** 2020-11-13

**Authors:** Jeanne Couturier, Léa Franconeri, Claire Janoir, Laurent Ferraris, Rabab Syed-Zaidi, Anlyata Youssouf, Cécile Gateau, Sandra Hoys, Julio Aires, Frédéric Barbut

**Affiliations:** 1 Faculty of Pharmacy, Paris University, INSERM UMR S-1139, 4 Avenue de l’Observatoire, 75006 Paris, France; laurent.ferraris@parisdescartes.fr (L.F.); julio.aires@u-paris.fr (J.A.); frederic.barbut@aphp.fr (F.B.); 2 National Reference Center for *Clostridioides difficile*, Saint-Antoine Hospital, 184 rue du Faubourg Saint-Antoine, 75012 Paris, France; lea.franconeri@hotmail.fr (L.F.); rabab.syedzaidi@aphp.fr (R.S.-Z.); anlyata.youssouf@aphp.fr (A.Y.); cecile.gateau@aphp.fr (C.G.); 3 Micalis Institute, INRA, AgroParisTech, Université Paris-Saclay, 92290 Châtenay-Malabry, France; claire.janoir-jouveshomme@universite-paris-saclay.fr (C.J.); sandra.hoys@u-psud.fr (S.H.)

**Keywords:** *Clostridioides difficile*, nontoxigenic strains, preterm neonates, *C. difficile* infection, colonization, in vivo model

## Abstract

In a previous monocentric study in preterm neonates (PN), we described a high *Clostridioides difficile* colonization rate (74%) with two uncommon non-toxigenic strains (NTCD) belonging to PCR-ribotype (RT) (CE)847 and (CE)032. To determine the extent of carriage of both NTCD in other spatio-temporal settings, strains isolated in PN stools from two multicenter cohorts were characterized by PCR-ribotyping, MLVA and MLST. We also evaluated the protective role of two NTCD from these RT against *C. difficile* infection in a hamster caecitis model. Animals were administered either each NTCD alone (n = 7), or followed by a 027 strain (n = 9). A control group received only the 027 strain (n = 8). Clinical activity and colonization by *C. difficile* in stools were monitored daily until death or sacrifice at D20. We isolated 18 RT(CE)032 (ST-83) strains and 2 RT(CE)847 (ST-26) strains among 247 PN from both cohorts. Within each RT, strains were genetically related. The survival rate was significantly increased when animals received a RT(CE)847 or (CE)032 strain before the 027 strain (4/9 deaths, *p* = 0.029; 1/9 death, *p* = 0.0004, respectively). We describe two predominant uncommon NTCD strains, in a PN population from different healthcare facilities. Both NTCD provide a potential protection against *C. difficile* infection.

## 1. Introduction

*Clostridioides difficile* is a Gram-positive, anaerobic, spore-forming bacterium, responsible for 15–25% of all cases of antibiotic-associated diarrhea [1]. Clinical presentations of *C. difficile* infections (CDI) range from mild self-limited diarrhea to severe pseudomembranous colitis. Risk factors for CDI include host-related factors (e.g., advanced age, comorbidities, immunodepression), factors causing a gut dysbiosis (e.g., use of antibiotics, proton-pump inhibitors) and factors exposing to *C. difficile* spores (e.g., long hospital stay, contaminated environment) [2,3]. The CDI incidence has significantly increased for the past two decades, in healthcare facilities as well as in community settings [4]. The emergence and spread of a hypervirulent epidemic strain called 027/B-I/NAPI partly explains this epidemiological shift. *C. difficile* virulence is mainly based on the production of two potent toxins, TcdA and TcdB. Non-toxigenic *C. difficile* strains (NTCD) are considered as non-pathogenic due to a lack of toxins A and B genes [5].

The presence of a toxigenic *C. difficile* strain in the gut does not always lead to symptoms. Asymptomatic colonization rates are low (2.4–13%) in healthy adults [6,7], and higher (14–24%) in hospital settings [8,9,10,11,12,13], NTCD accounting for 17% to 40% of the strains. However, few studies have focused on NTCD, thus data regarding their characteristics and epidemiology remain scarce.

The primary prevention of CDI relies on appropriate use of antibiotics and prevention of cross-transmission [14]. The potential protection against CDI conferred by a previous colonization was suggested by epidemiological studies in hospitalized patients with a follow-up from admission to discharge [13]. A phase 2 controlled trial versus placebo demonstrated that colonization by the M3 NTCD strain prevented CDI recurrences in patients experiencing a first CDI episode or a first CDI recurrence [15]. However, the effect of NTCD colonization in preventing a first CDI episode has not been evaluated yet in humans.

Children under 2 years of age are commonly asymptomatically colonized with toxigenic or non-toxigenic *C. difficile* strains, with rates ranging from 2% to 70% [16,17]. Preterm neonates (PN) are at higher risk for *C. difficile* colonization than full-term neonates, due to a frequent antibiotic administration and longer hospital stays. In a previous monocentric prospective cohort study [18], we showed that up to 74% of preterm neonates were colonized mostly by NTCD. We identified two predominant NTCD belonging to [CE(capillary-electrophoresis)]032 and (CE)847 RTs, representing 78% of all strains isolated during the study period. MLVA typing showed that within both RTs, strains belonged to the same clone or were genetically related, suggesting a cross-transmission in the healthcare facility. We also observed that only few PN (7%, 8/121) were colonized by *C. difficile* toxigenic strains, suggesting that the predominant NTCD isolated in our PN cohort could prevent the colonization by toxigenic strains.

In the present study, our objectives were to characterize the *C. difficile* strains isolated in three cohorts of PN by MLVA and MLST and to determine the potential protective effect of the two predominant NTCD [(CE)032 and (CE)847] in the Golden Syrian hamster model of acute CDI.

## 2. Materials and Methods

### 2.1. Phenotypic and Genotypic Characteristics of C. difficile Strains Isolated in Three Cohorts of Preterm Neonates

#### 2.1.1. Description of the Cohorts

The PREMAFLORA study focused on describing the *C. difficile* carriage and colonization dynamics among 121 PN [18]. It was a one-year (2008–2009) longitudinal monocentric prospective study.

The ClosNEC cohort aimed to study the relationship between *Clostridium* colonization and the development of necrotizing enterocolitis (NEC) in PN [19]. It was a 16-month (2015–2016) multicenter prospective study including 159 PN from 20 neonatal intensive care units (NICUs).

The EPIFLORE study aimed to assess the relationship between feeding strategies, intestinal microbiota composition and the development of NEC in PN [20]. It was a one-year (2011) multicenter prospective study. Stool samples were obtained and cultivated for 88 PN, coming from 20 NICUs.

#### 2.1.2. *C. difficile* Isolation and Strain Characterization

All stool samples were processed as described by Ferraris et al. [18]. Briefly, fecal samples were spread on *C. difficile* selective medium CLO-M (bioMérieux^®^, Marcy-l’Etoile, France) and incubated for 24 h at 37 °C under anaerobic conditions (CO_2_:H_2_:N_2_, 10:10:80, anaerobic chamber). Isolates were stored at −80 °C in BHI (brain heart infusion) liquid media supplemented with 15% glycerol. Isolates identification was confirmed by PCR amplification and sequencing of the 16S ADNr gene and comparison with the NCBI Blast^®^ database (https://blast.ncbi.nlm.nih.gov/Blast.cgi accessed December 2017). *C. difficile tpi, tcdA,* and *tcdB* genes were screened by multiplex PCR, as previously described [21].

#### 2.1.3. PCR-Ribotyping

The PCR-ribotype (RT) of 199 strains was previously described in the PREMAFLORA study by Ferraris et al. [18]. We determined the RT of 37 strains from the ClosNEC cohort and 12 strains from the EPIFLORE cohort as previously described [22], using a capillary gel-based electrophoresis. Briefly, after DNA extraction and amplification, a mix of 1 µL of a 1/10 dilution of each PCR product, 10.5 µL formamide and 0.5 µL GeneScan LIZ600 (Applied Biosystems, Foster City, CA, USA) was denatured for 30 s at 90 °C. Capillary electrophoresis was performed on 8-capillary 3500 Genetic Analyzer (Applied Biosystems). The profiles were analyzed with GeneMapper software (Thermo Fischer Scientific, Villebon-sur-Yvette, France). Strains were assigned a RT with either the standard nomenclature when available (WEBRIBO database, https://webribo.ages.at/ accessed February 2018), or the specific nomenclature of the French National Reference Laboratory for *C. difficile*.

#### 2.1.4. Multi-Locus Variable-Number Tandem-Reapeat Analysis (MLVA)

MLVA typing data were previously obtained for 199 strains in the PREMAFLORA study [18]. We additionally performed MLVA typing for 37 strains from the ClosNEC cohort and 12 strains from the EPIFLORE cohort. A seven-VNTR scheme (A6, B7, C6, E7, F3, G8, H9) was used, as previously described [23]. The number of tandem repeats was determined by capillary gel-based electrophoresis and concatenated to generate a MLVA-type, using BioNumerics software (Applied Maths NV, Sint-Martens-Latem, Belgium). The genetic distance between two strains was determined by calculating the STRD (summed tandem-repeat differences, i.e., the sum of the number of differences at the 7 loci). Strains with a STRD ≤ 10 were genetically related and they were part of the same clonal complex if the STRD was ≤2. Typing data were represented with a minimum spanning tree using the Manhattan coefficient.

#### 2.1.5. Multi-Locus Sequence Typing (MLST)

For MLST analysis, we selected 59 strains representative of each MLVA-type and 5 strains of each predominant RT [(CE)032 and (CE)847] (n = 47 for PREMAFLORA, n = 13 for ClosNEC, n = 9 for EPIFLORE). Seven housekeeping genes (i.e., *adk, atpA, dxr, glyA, recA, sodA* and *tpi*) were amplified and sequenced as previously described [24]. The sequence type (ST) was obtained by comparing the sequences with the database (https://pubmlst.org/cdifficile/ accessed June 2019). Sequence profiles were analyzed with the S.T.A.R.T.2 software [25].

### 2.2. In Vivo Study of NTCD Properties to Prevent C. difficile Infection

#### 2.2.1. *C. difficile* Strain and Spore Preparation

The toxigenic RT 027 strain (CD13-125) produced toxins A and B as well as an additional toxin called the binary toxin. This strain was isolated in the stools of a CDI patient hospitalized in a French healthcare facility in 2013 [26]. We selected two NTCD from each RT previously identified as predominant in the PREMAFLORA cohort: strains PCD130 (RT (CE)847) and PCD182 (RT (CE)032) [18].

After an overnight culture at 37 °C under anaerobic conditions on *C. difficile* selective medium CLO-M (bioMérieux^®^), colonies were plated on a Columbia medium with 5% horse blood and incubated for 48 h at 37 °C under anaerobic conditions. Plates were left at room temperature for 5 days under aerobic conditions to stimulate the spore production. The bacterial culture was then suspended in 2 mL of sterile water and an alcoholic shock (absolute ethanol *v*/*v*, 30 min at 25 °C) was performed to kill remaining vegetative cells. Spores were centrifuged (3000 rpm, 20 min) and re-suspended in 1 mL of absolute ethanol. After 1 min of sonication (35 MZ Sonorex SHE 10000, Bandelin^®^, Berlin, Germany), the presence of spores was confirmed by direct microscopic examination. After two washes in NaCl 0.9% and centrifugation (6000 rpm, 5 min), the pellet was re-suspended in 700 µL of NaCl 0.9%. Colonies were enumerated on ChromID *C. difficile* (bioMérieux^®^) media to determine the final spore concentration and the volume to be administered.

#### 2.2.2. Animal Study Design

Adult Syrian golden hamsters were used for the study. Forty females (8 weeks old, weight ranging from 80 to 110 g) were housed in individual cages fitted with covers holding disposable polyester air filters in an animal biosafety level 2 facility within the Central Animal Facility of the Pharmacy Faculty, according to European Union guidelines for the handling of laboratory animals. Procedures for infection, euthanasia and specimen collection were approved by the ethical Committee CAPSUD (APAFIS 7492-2016101014285698). Absence of *C. difficile* colonization was assessed before the beginning of the study by cultivating the stools on BHI agar medium supplemented with 0.1% taurocholate and selective *C. difficile* supplement as recommended by the manufacturer (Oxoid, Basingstoke, UK).

**Figure 1:**
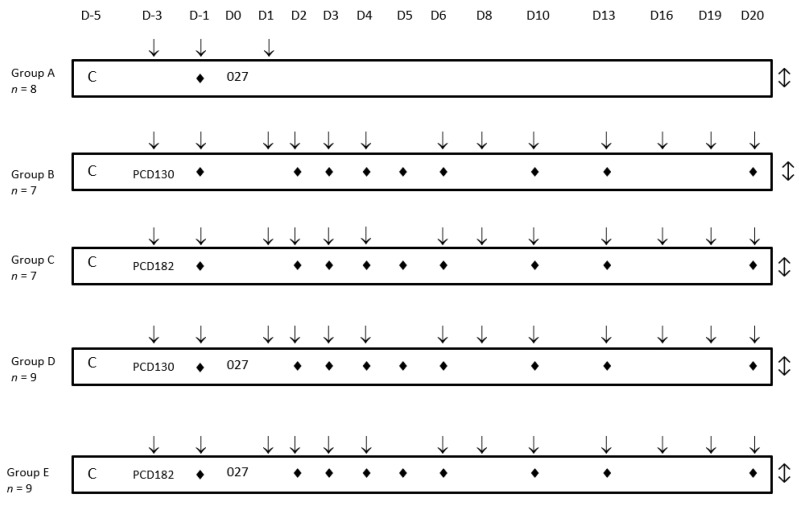
Experimental design of the In vivo study. C: Clindamycin administration; ♦: stool sampling; ↓: weighing; ↕: euthanasia of surviving hamsters.

The study design is shown in Figure 1. To establish an intestinal dysbiosis, animals received a single dose of 50 mg/kg of clindamycin by oral gavage at D-5 [27]. *C. difficile* strains were administered according to 5 groups: group A (n = 8) received the toxigenic RT027 strain at D0; groups B (n = 7) and C (n = 7) received, respectively, NTCD strains PCD130 (RT (CE)847) and PCD182 (RT (CE)032) at D-3; groups D (n = 9) and E (n = 9) received, respectively, PCD130 and PCD182 at D-3 followed by the administration of the RT027 strain at D0. Hamsters were given 5 × 10^3^ spores of RT027 strain and 1 × 10^6^ spores of NTCD by oral challenge. A daily monitoring using a clinical activity score (Table 1) was implemented from D-3 until the end of the experiment at D20. Stool samples collection and regular weighing were performed from D-3. Animals presenting CDI clinical signs, a weight loss >20% and/or a clinical activity score of 0 were sacrificed. Surviving animals at D20 were sacrificed. Caeca were sampled, washed and stored in a formol-PBS 4% solution for 24 h, then in 70% ethanol (+4 °C) before histology analysis. After paraffin inclusion and microtome cut (3 µm), slides were colored with hematoxylin and eosin before scan and analysis (3DHISTECH Slide Converter^®^ software). Histological analyses were performed by the platform of the National Institute of Agronomical Research (INRA, Jouy-en-Josas, France).

#### 2.2.3. Colonization Kinetics and Bacterial Counts

Stools were weighted and suspended in 1 mL PBS. After homogenization, 500 µL were treated by alcoholic shock as previously described. Untreated and treated suspensions were enumerated by inoculating 100 µL of serial 1/10 dilutions on ChromID *C. difficile* medium to determine total bacteria and spore counts, respectively. For groups D and E, a differential enumeration was performed using a BHI agar medium supplemented with 1 g/L taurocholate, 250 mg/L cycloserine, 8 mg/L cefoxitine and 2 mg/L erythromycin. This medium allowed the selective growth of the erythromycin resistant strain RT027, as opposed to strains PCD130 (RT (CE) 847) and PCD182 (RT (CE)032). To confirm strain identification in the animals’ stools and exclude cross-contamination, 2 to 5 isolates per animal were characterized by PCR-ribotyping as described above. Results were expressed in CFU/g of stools.

### 2.3. Statistical Analysis

Bacterial counts were expressed by their means and standard deviations. The survival rates of the treated groups at D20 were compared with an exact Fisher test. Survival curves were compared between groups by a log-rank test, with the Benjamini–Hochberg correction or False Discovery Rate (FDR) (survival package, Rstudio^®^).

## 3. Results

### 3.1. Typing Data Show that Predominant NTCD in Preterm Neonates Are Genetically Related

PCR-ribotyping results were previously reported for the PREMAFLORA cohort for 199 strains [18]. In the ClosNEC cohort, of the 159 stool samples analyzed by culture, 37 (23%) were *C. difficile* positive. Thirty-five strains (95%) were NTCD including 14 (CE)032 (38%). The other NTCD belonged to RT 010 (*n* = 11), 140 (*n* = 7), (CE)151 (*n* = 2), and (CE)338 (*n* = 1). No (CE)847 strain was isolated. Only two toxigenic strains were recovered (one (CE)236 and one with no matching profile in the WEBRIBO RT database). In the EPIFLORE cohort, 12 (14%) out of 88 stool samples were *C. difficile* positive. Among the 12 colonized neonates, 2 (17%) were colonized by a (CE)847 strain, and 4 (33%) by a (CE)032 strain. The other NTCD belonged to RT 140 (*n* = 3) and (CE)031 (*n* = 1). Two neonates were colonized by a toxigenic RT 014 strain.

**Figure 2:**
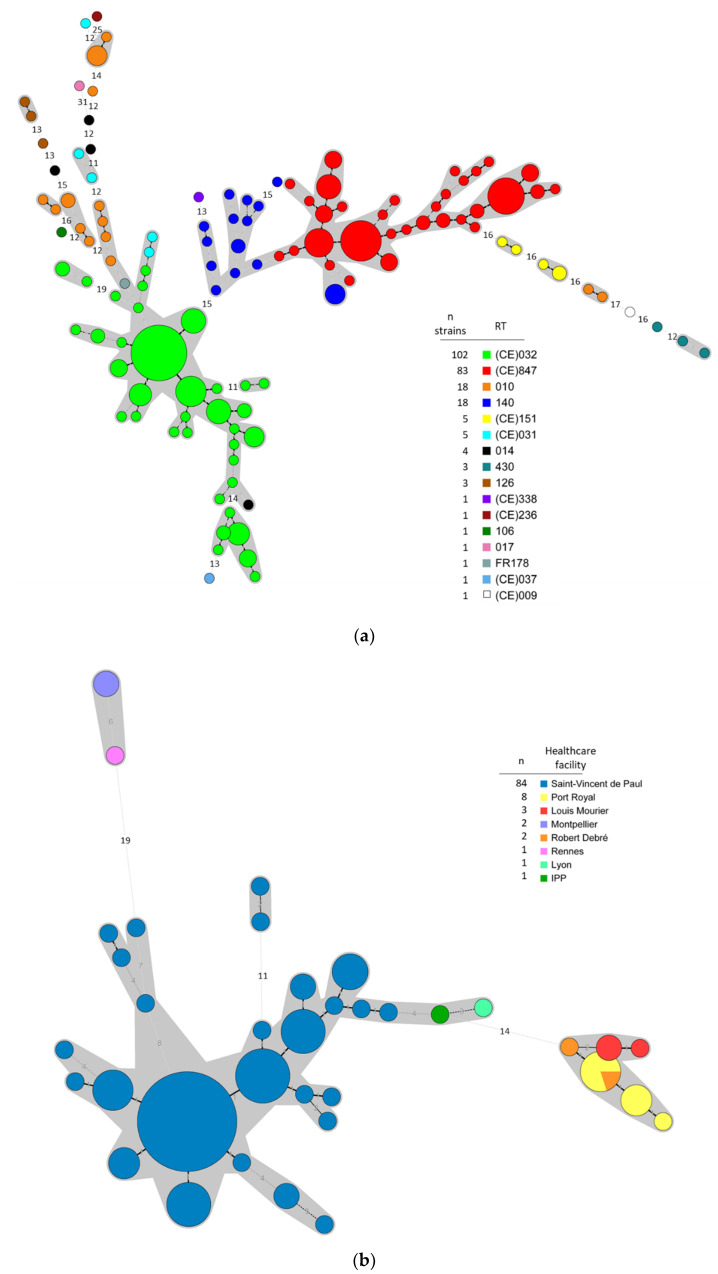
Minimum spanning tree representations of Multi-Locus Variable number tandem-repeat Analysis (MLVA) data of the 248 *C. difficile* isolates (**a**) and the 102 (CE)032 isolates (**b**). The circles represent unique MLVA-types and are scaled to member count. The number between the circles represents the STRDs (summed tandem repeat differences) between MLVA-types. Grey areas represent genetically related MLVA-types (STRD ≤ 10) while MLVA-types linked by a plain line are part of the same clonal complex (STRD ≤ 2). MLVA-types are color-coded according to the RT for the (**a**) and according to the healthcare facility for the (**b**). IPP: Paris child care institute.

MLVA typing data for 248 strains (*n* = 199 for PREMAFLORA, *n* = 37 for ClosNEC, *n* = 12 for EPIFLORE) were concatenated in a minimum spanning tree and represented in the Figure 2a. MLVA analysis revealed two major clusters (STRD ≤ 10), one comprising all (CE)847 strains (*n* = 83) and almost all RT 140 strains (17/18), and the other comprising most (CE)032 strains (84/102), 1 RT 014, 1 FR178, 2 (CE)031, and 4 RT 010 strains. MLVA data targeted on the 102 (CE)032 strains are represented in Figure 2b. Almost all (82/84) strains isolated in Saint-Vincent-de-Paul NICU were part of the same cluster, the remaining two strains form a clonal complex and are very close to the main cluster (STRD = 11). All strains isolated in Parisian NICUs in 2015–2016 (Port-Royal, n = 8; Louis Mourier, *n* = 3; Robert Debré, *n* = 2) are part of the same clonal complex (STRD ≤ 2). Three strains isolated in other French areas (Montpellier, *n* = 2; Rennes, *n* = 1) in 2011 form another cluster.

**Figure 3:**
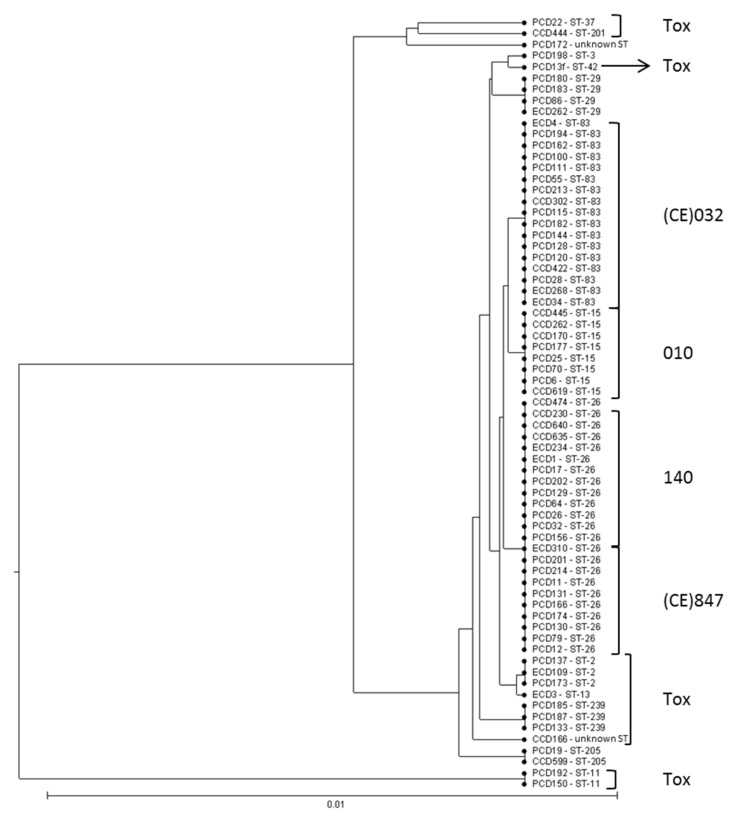
Tree representation of MLST data for 69 *C. difficile* strains with the UPGMA algorithm. The distance matrix was constructed using sequence profile data. Tox: toxigenic strains.

The Unweighted Pair Group Method with Arithmetic Mean (UPGMA) tree representation from sequence profiles data of 69 strains is shown in Figure 3. All RT 140 (*n* = 12) and (CE)847 (*n* = 10) strains belonged to ST-26 and were part of the same cluster as all (CE)032 strains (*n* = 17, ST-83) and RT 010 strains (*n* = 8, ST-15).

### 3.2. NTCD Have A Protective Effect against C. difficile Colonization and Infection

**Figure 4:**
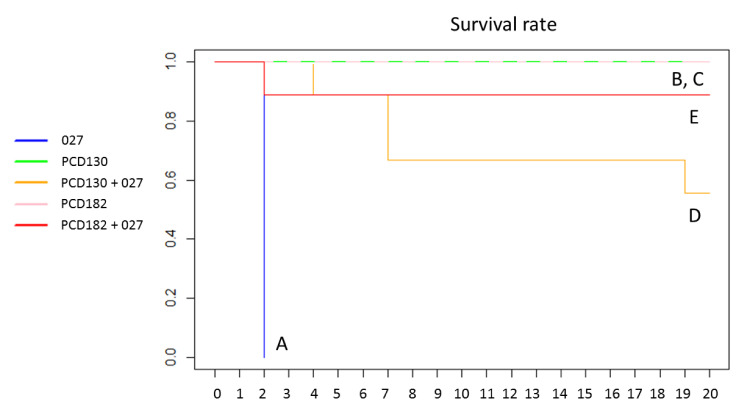
Survival curve of the five hamsters groups over time.

The survival curve of the five animal groups is represented in the Figure 4. In the positive control group (A), infection by the RT027 strain resulted in a 100% mortality rate at D2. No animal died in the negative control groups B (PCD130) and C (PCD182). When animals were pre-treated with PCD130 (group D) or PCD182 (group E) before challenge with the RT027 strain, the mortality rate at D20 decreased to 44% (4/9 hamsters) and 11% (1/9 hamsters), respectively. In the group D, 4 hamsters were euthanized due to a low clinical activity score and a significant weight loss (approx. 20%). The only deceased hamster in the group E died before any clinical sign could have been detected. The survival rate at D20 significantly increased when animals were administered PCD130 (group D, *p* = 0.029) or PCD182 (group E, *p* = 0.0004) before the toxigenic strain, compared to the positive control group A. There was no significant difference between survival rates at D20 in groups D and E (*p* = 0.29). The log rank test showed that the survival probability differed significantly between co-infected groups D and E and the control group A (*p* = 6.10^−5^ for both groups).

**Figure 5:**
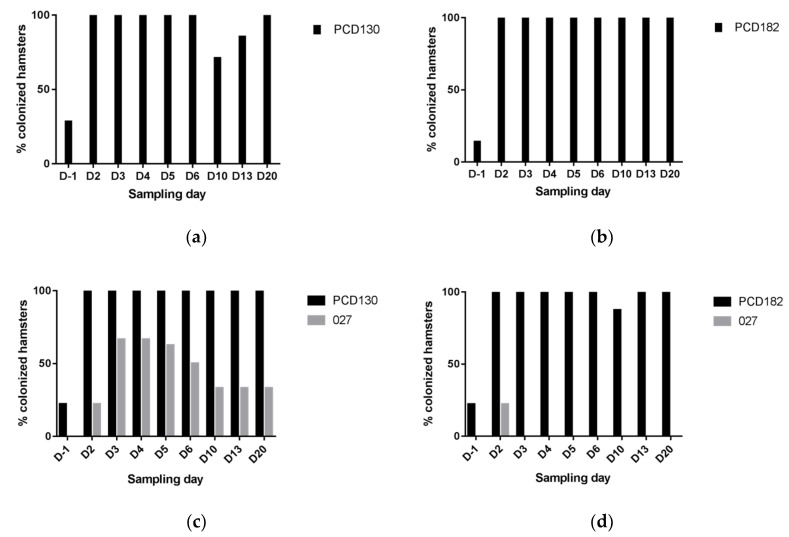
Colonization rates among surviving hamsters in the PCD130 (**a**), PCD182 (**b**), PCD130 + 027 (**c**), and PCD182 + 027 (**d**) groups (respectively B, C, D and E).

**Figure 6:**
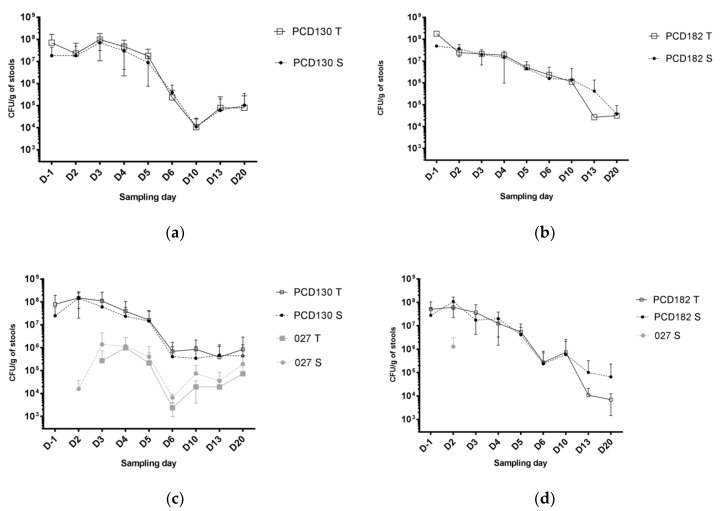
Mean colonization levels over time among colonized hamsters in the PCD130 (**a**), PCD182 (**b**), PCD130 + 027 (**c**) and PCD182 + 027 (**d**) groups (respectively B, C, D and E). T: total forms; S: sporulated forms. Error bars represent the standard deviation. The value for the group C at D-1 (**b**) is the observed count for the only colonized animal, therefore no error bar is represented.

The mean colonization level by the RT027 strain in group A at D2 was 1.6 ± 1.7 × 10^7^ CFU/g of stools, of which 93% were sporulated forms. Two days after administration (D-1), NTCD colonization rates for groups B, C, D and E were low (28.6%, 14.3%, 22.2%, and 22.2% of hamsters, respectively) (Figure 5). Mean NTCD colonization levels among colonized hamsters for groups B, C, D and E were 7.0 ± 9.9 × 10^7^, 1.8 × 10^8^, 8.0 ± 11.3 × 10^7^ and 5.1 ± 5.5 × 10^7^ CFU/g of stools, respectively (Figure 6). Five days after administration (D2), all animals of groups B, C, D, and E became colonized by NTCD (Figure 5). Mean NTCD colonization levels at D2 in animals treated by PCD130 were 2.3 ± 4.5 × 10^7^ (group B) and 1.5 ± 0.9 × 10^8^ (group D) CFU/g of stools, with a sporulated population of 81% and 98%, respectively. For groups C and E, treated by PCD182, mean NTCD colonization levels at D2 were 2.4 ± 1.2 × 10^7^ and 6.2 ± 4.0 × 10^7^ CFU/g of stools, respectively, with 100% of sporulated forms. In groups B, C, D and E, mean NTCD colonization levels steadily decreased between D2 and D20 to reach 7.9 ± 19.6 × 10^4^, 3.2 ± 3.3 × 10^4^, 8.5 ± 21 × 10^5^ and 6.9 ± 5.5 × 10^3^ CFU/g of stools, respectively.

Within group D (*n* = 9), three hamsters remained 027-free throughout the experiment. Two hamsters were colonized by the toxigenic strain from D2 until death at D7 (*n* = 1), or until the end of the experiment without any CDI sign (*n* = 1). The toxigenic strain was detected in three other hamsters from D3 until death at D4, D7 and D19. Finally, one animal was co-colonized only from D3 to D6 and did not show any CDI sign. In this group, mean levels of colonization by the toxigenic strain were comprised between 10^3^ and 10^6^ CFU/g of stools throughout the experiment.

In the group E (*n* = 9), the toxigenic strain was only found in two hamsters at D2 under sporulated form (1.3 ± 1.8 × 10^6^ CFU/g of stool), leading to the death of one animal, the other becoming 027-free from D3 until the end of the experiment. No toxigenic strain was isolated in the stool samples of the seven other animals from D2 until D20.

**Figure 7:**
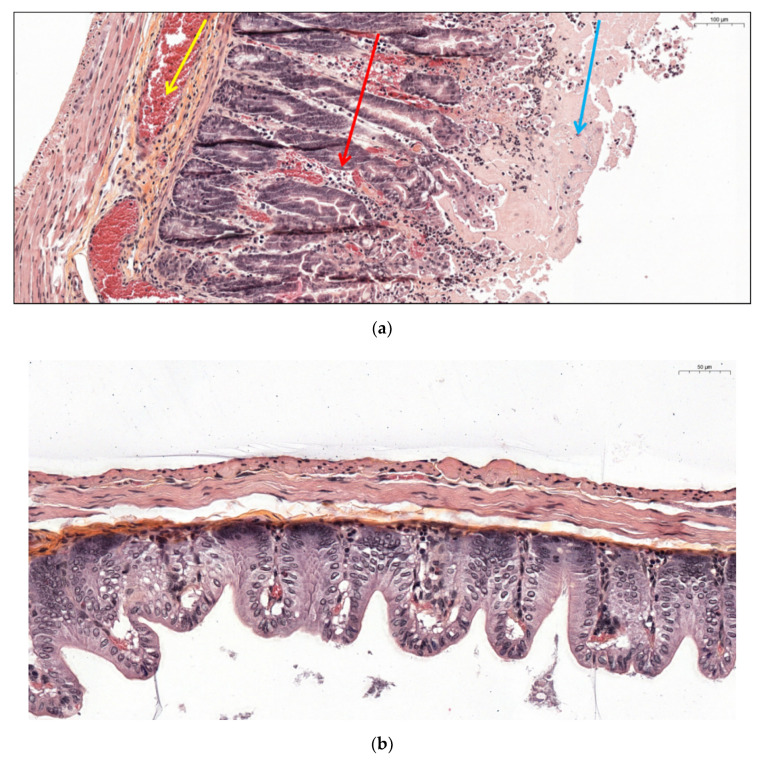
(**a**) Caecal histological section from a hamster co-infected by PCD130 + 027 (group D) euthanized after presenting CDI clinical signs. Diffuse hemorrhage is designated by the yellow arrow, polynuclear infiltration by the red arrow, hyperplasia and epithelial desquamation by the blue arrow. (**b**) Intestinal histological section from a hamster colonized by PCD130 (group B) surviving at D20.

The typical hemorrhagic and hyperplasic aspect of organs harvested in euthanized hamsters due to clinical signs of CDI is shown in Figure 7a. No tissular alteration was observed in animals who received the NTCD alone (groups B and C), as well as co-infected animals (groups D and E) without CDI signs (Figure 7b). In co-infected hamsters euthanized after clinical signs of CDI (group D), we observed important caecal tissular alterations with diffuse hemorrhage, infiltration by pro-inflammatory cells (neutrophils), cryptic intestinal architecture loss, hyperplasia and epithelial desquamation (Figure 7a). Caeca of animals in the control group A and of the deceased hamster in the group E could not be harvested due to tissue stiffness.

The animal weight increased in all groups, except for hamsters of the positive control group A and co-infected hamsters with clinical CDI signs and precociously deceased (Figure S1).

## 4. Discussion

Using a monocentric longitudinal PN cohort (n = 121), we previously observed a high *C. difficile* colonization rate (up to 74%), mostly by NTCD [18]. Two clonal NTCD belonging to RT (CE)032 and (CE)847 were identified, representing 78% of isolated strains. In this study, additional data were collected by characterizing *C. difficile* strains from two other multicentric PN cohorts (ClosNEC and EPIFLORE). The colonization rates for ClosNEC and EPIFLORE (23%, and 14%, respectively) were lower compared to PREMAFLORA colonization rate and to another study reporting that 55% of neonates in intensive care units were colonized [28]. Most of the strains were NTCD, as previously described among 0–2 years old children in a French healthcare facility [29].

To complement the data obtained during our first study, all strains isolated in ClosNEC and EPIFLORE were characterized by MLVA; MLST was performed on a subset of 69 strains representative of each MLVA-type from the three cohorts. Overall, typing data (MLVA, MLST) showed that both RT (CE)032 and (CE)847 were closely related to each other and to other well-described NTCD RT such as 140 [30] and 010 [31]. We confirmed that RT (CE)032 and (CE)847 frequently colonized the PN’s gut. In particular, (CE)032 strains were isolated several years apart in PN coming from 8 healthcare facilities in France, suggesting an improved ability to adapt to the PN’s gut microbiota and/or to survive in the NICU environment.

The persistence of NTCD may stem from specific factors, or selective advantages allowing the occupation of the ecological niche. Concerning the antimicrobial susceptibility patterns, a recent work reported a higher resistance to MTZ in NTCD compared to toxigenic strains [32]. We did not confirm this finding since no MTZ MIC was above 0.75 mg/L (Table S1). In 2017, MLVA analysis of hypervirulent 027 strains showed that the same clone could be isolated in adult patients hospitalized years apart in the same healthcare facility [23]. The resistance to ERY and MXF commonly described in epidemic 027 strains is a potential explanation for its persistence. In our study, the only common antibiotic resistance trait observed for (CE)032 and (CE)847 strains was RIF resistance (Table S1), an antibiotic commonly used to treat staphylococcal infections, particularly neonatal bacteremia [33].

Following the identification of the two predominant (CE)032 and (CE)847 RT in PN, we tested In vivo their potential to prevent CDI due to a hypervirulent 027 strain, using the hamster model of caecitis. The selected strains were both resistant to clindamycin, the antibiotic used to induce dysbiosis in our animal model. The rate of fatal CDI dropped from 100% in the control group, infected by the RT027 strain, to 11% and 44% if animals were previously administered PCD182 (RT (CE) 032) or PCD130 (RT (CE)847), respectively. PCD182 (group E) successfully inhibited the colonization by the RT027 strain until D20, since the toxigenic strain was only isolated in stools of 2/9 hamsters, and only at one sampling time (D2). From D3 to D20, no toxigenic strain was isolated in stool samples of animals above the detection limit (10^3^ CFU/g of stools). The inhibitor effect of PCD130 was lower, RT027 levels in stools being low (10^3^ to 10^6^ CFU/g of stools) but detectable throughout the experiment. These results are consistent with the higher mortality rate in the PCD130-treated group. Variable protective effects of NTCD were previously reported (34–36). Our results support these findings, although the survival rate at D20 did not significantly differ between both NTCD (*p* = 0.29), probably because of a lack of statistical power due to a limited number of animals in each group. We observed declining NTCD colonization levels for all groups throughout the experiment, probably related to the restoration of the normal gut microbiota of hamsters.

Several In vivo experiments in models of hamsters treated by clindamycin showed that prior colonization by NTCD could confer a protection against toxigenic strains [27,34,35,36]. Sambol et al. observed a protection ranging from 87 to 97% with three NTCD frequently isolated in patients, the M3 strain being the most effective [36]. To date, only the M3 and T7 NTCD strains, isolated in adult patients, were effective against CDI due to epidemic RT027 hypervirulent strain [27]. The M3 strain reduced the incidence of fatal CDI in animals from 92% to 10%, a protection level similar to that observed in our study with PCD182 (from 100% to 11%). The T7 strain only reduced the incidence of fatal CDI from 92% to 40%, which is consistent with the results obtained with PCD130 (from 100% to 44%).

Our observations suggest that PCD182 could efficiently prevent the toxigenic strain from colonizing the gut since only two animals were co-colonized. Among them, one animal rapidly deceased at D2, and the other cleared the toxigenic strain at D3. In contrast, among nine hamsters treated by PCD130, six were co-colonized by the toxigenic strain, including four deceased. In the study by Nagaro et al. [27], only 1/10 hamster, uncolonized by the M3 strain, was shown to be colonized by the epidemic RT027 strain and died at D7. Conversely, 5/10 hamsters were co-colonized by the T7 strain and the RT027 strain, four of which died before D20. Taken together, these results suggest that when a co-colonization occurs, most animals develop a CDI (5/8 in both groups D and E in our study). The rapid and efficient gut colonization by a NTCD strain therefore appears essential to prevent colonization by the toxigenic strain and subsequent fatal CDI in hamsters. In our study, the NTCD colonization rate reached 100% of animals only five days after administration, slower than the M3 strain (90% of colonization in one day, 100% in three days) [27].

Bacterial enumeration showed that *C. difficile* strains were mostly present under sporulated form in hamster’s stools. These findings are consistent with current knowledge about CDI transmission, i.e., *C. difficile* excretion in the stools consisting mostly of spores [37]. However, our sampling process could partly explain the high rate of spores since stools were collected in the cages. Samples might have remained several hours to ambient air after passing, triggering the sporulation process.

There are several limitations to our study. First, the size of our groups (*n* < 10) reduces the statistical power of our experiments, impairing the possible demonstration of a significant correlation between colonization levels by the RT027 strain and death rates. Second, coprophagia of hamsters could be a source of recontamination by the toxigenic strain, despite daily litter changes. Third, because all animals were sacrificed at D20, the persistence of the protective effect of NTCD colonization needs to be confirmed. However, our results showed that 100% of surviving animals were still colonized by NTCD at the end of the experiment, despite mean levels lower than at D2. A persistent protection by the M3 strain up to 150 days was observed by Sambol et al. [36].

In conclusion, we showed that two specific NTCD RT were predominant among PN in various spatio-temporal settings. In vivo, we showed that two NTCD from representative RT were able to prevent the colonization by a toxigenic strain and to drastically reduce the mortality rate of CDI in the hamster model of acute infection. We hypothesized that those strains could be promising candidates for primary prevention of CDI. To better understand the In vivo behavior of both NTCD, further in vitro experiments would be necessary. Moreover, whole-genome sequencing of both strains would enable to identify genetic determinants responsible for the enhanced colonization abilities observed in our study. Clinical trials are now necessary in order to demonstrate their safety and efficacy in humans.

## Figures and Tables

**Table 1:** Clinical activity score.

Activity Score	Symptoms
0 (animals to euthanize)	Immobility, bradypnea, swollen stomach, wet tail and legs, squinted eyes, bristled hair, areflexia, weight loss >20%
1	Motor retardation, trembling, swollen stomach, tachypnea, bristled hair, reaction to external stimuli, wet tail, isolation, weight loss >10% and <20%
2	Normal activity

## Supplementary Materials

The following are available online at https://www.mdpi.com/2077-0383/9/11/3650/s1, Table S1: antibiotic susceptibility testing, Figure S1: Evolution of hamster’s weight over time.

## Patents

J.A., L.F. (Léa Franconeri) and F.B. are curently applying for a patent (patent application) no. FR2001857 relating to the content of the manuscript.
