# Supplementary material for: Characterization of Non-Toxigenic Clostridioides difficile Strains Isolated from Preterm Neonates and In Vivo Study of Their Protective Effect
Source: J Clin Med. 2020 Nov 13;9(11):3650. doi: 10.3390/jcm9113650 (PMC7696784; doi:10.3390/jcm9113650)

## Supplementary materials

### Supplementary Table S1: Antimicrobial susceptibility testing

Antimicrobial susceptibility was assessed for the subset of 69 strains typed by MLVA and MLST. Susceptibility to erythromycin (ERY), clindamycin (CM), moxifloxacin (MXF), tetracycline (TET), rifampicin (RIF), chloramphenicol (CL), imipenem (IMI) and amoxicillin (AMX) was determined using the disk diffusion method. Briefly, a 2 McF strain suspension was inoculated on Brucella Agar with 5 % horse blood (Becton Dickinson, Franklin Lakes, New Jersey, United States). The MICs to metronidazole (MTZ) and vancomycin (VA) were determined using E-test® strips (bioMérieux®). Plates were incubated 48 h at 35-37°C and diameters were interpreted according to the criteria for anaerobic bacteria of the CA-SFM (Comité de l'Antibiogramme de la Société Française de Microbiologie) and the EUCAST (European Committee on Antimicrobial Susceptibility Testing) (https://www.sfm-microbiologie.org/2020/04/07/casfm-eucast-v1-0-avril-2020/). Resistance breakpoints were set as follows: ERY (15 UI)<15 mm ; CM (2 UI)<15 mm ; MXF (5 µg) <18 mm; TET (30 µg)<19 mm ; RIF (30 µg)<14 mm ; CL (30 µg)<15 mm; IMI (10 µg)<14 mm ; AMX (20 µg)<17 mm. For MTZ and VA, a reduced susceptibility was defined as MIC>4 mg/L and >2 mg/L, respectively.

All 69 strains were resistant to CM, and susceptible to IMI, AMX, MTZ, and VA. All VA and MTZ MICs were ≤1 mg/L and ≤0.75 mg/L, respectively. The susceptibility testing results for TET, ERY, RIF, MXF and CL are shown in the Table S1.

**Table S1.** Antimicrobial susceptibility testing results for TET, ERY, RIF, MXF and CL

| RT (n) | TET | ERY | RIF | MXF | CL |
|---|---|---|---|---|---|
| (CE)032 (17) | S (17) | S (17) | **R (17)** | S (17) | S (16)/**R (1)** |
| 140 (12) | S (12) | S (9)/**R (3)** | S (5)/**R (7)** | S (12) | S (12) |
| (CE)847 (10) | S (10) | S (8)/**R (2)** | **R (10)** | S (10) | S (10) |
| 010 (8) | S (8) | S (4)/**R (4)** | S (8) | S (7)/**R (1)** | S (8) |
| 014* (4) | S (4) | S (4) | S (4) | S (4) | S (4) |
| (CE)031 (4) | S (4) | S (4) | S (4) | S (4) | S (4) |
| 430* (3) | S (3) | S (3) | S (3) | S (3) | S (3) |
| (CE)151 (2) | S (2) | S (2) | S (2) | S (2) | S (2) |
| 126* (2) | **R (2)** | S (2) | S (2) | S (2) | S (2) |
| 017* (1) | S (1) | **R (1)** | S (1) | S (1) | S (1) |
| (CE)009 (1) | S (1) | **R (1)** | S (1) | S (1) | S (1) |
| 106* (1) | S (1) | S (1) | S (1) | S (1) | S (1) |
| (CE)338 (1) | S (1) | **R (1)** | S (1) | **R (1)** | S (1) |
| FR178* (1) | S (1) | S (1) | S (1) | S (1) | S (1) |
| (CE)236* (1) | S (1) | S (1) | S (1) | S (1) | S (1) |
| (CE)037 (1) | S (1) | S (1) | S (1) | S (1) | S (1) |

* Toxigenic strains; S: susceptible; R: Resistant

**Supplementary Figure S1 :** Evolution of hamster's weight over time.

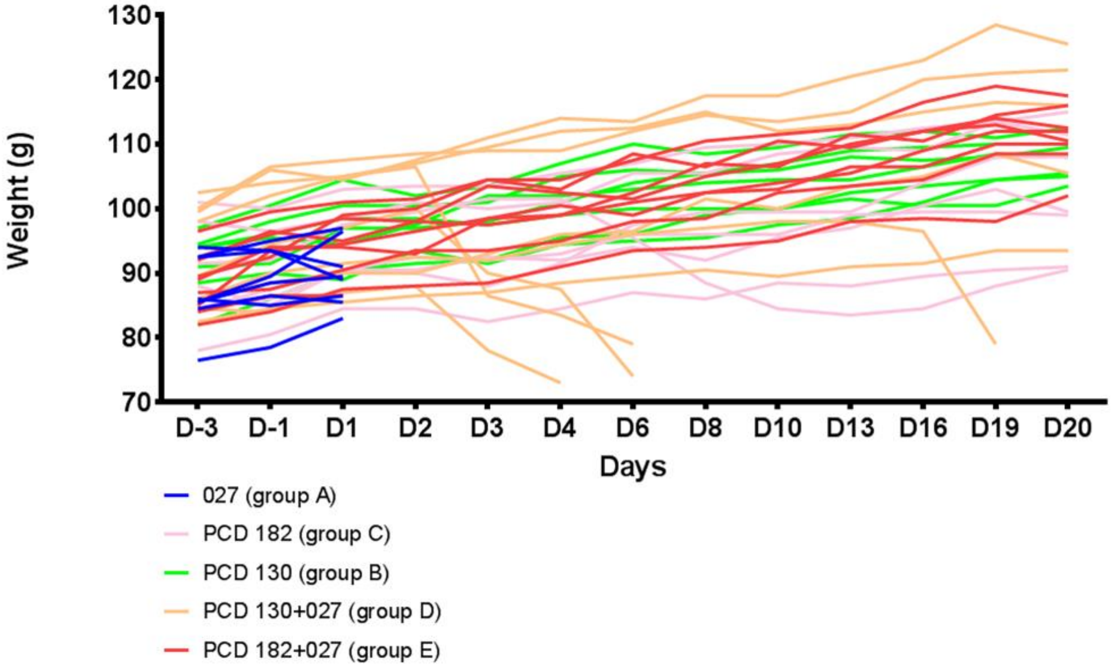

Supplement: Supplementary file 1 [file jcm-09-03650-s001.pdf]
